# Identification of antimycin A as a c-Myc degradation accelerator *via* high-throughput screening

**DOI:** 10.1016/j.jbc.2023.105083

**Published:** 2023-07-24

**Authors:** Ziyu Liu, Kosuke Ishikawa, Emiko Sanada, Kentaro Semba, Jiang Li, Xiaomeng Li, Hiroyuki Osada, Nobumoto Watanabe

**Affiliations:** 1 Bioprobe Application Research Unit, RIKEN CSRS, Wako, Saitama, Japan; 2 Graduate School of Medical and Dental Sciences, Tokyo Medical and Dental University, Bunkyo-ku, Tokyo, Japan; 3 Japan Biological Informatics Consortium (JBiC), Koto-ku, Tokyo, Japan; 4 Chemical Biology Research Group, RIKEN CSRS, Wako, Saitama, Japan; 5 Chemical Resource Development Research Unit, RIKEN CSRS, Wako, Saitama, Japan; 6 Department of Life Science and Medical Bioscience, School of Advanced Science and Engineering, Waseda University, Shinjuku-ku, Tokyo, Japan; 7 Medical-Industrial Translational Research Center, Fukushima Medical University, Fukushima, Japan; 8 Guangdong Engineering Research Center of Oral Restoration and Reconstruction, Affiliated Stomatology Hospital of Guangzhou Medical University, Guangzhou, Guangdong, China; 9 Department of Pharmaceutical Sciences, University of Shizuoka, Suruga-ku, Shizuoka, Japan

**Keywords:** c-Myc, ubiquitin, phosphorylation, degradation, reactive oxygen species

## Abstract

c-Myc is a critical regulator of cell proliferation and growth. Elevated levels of c-Myc cause transcriptional amplification, leading to various types of cancers. Small molecules that specifically inhibit c-Myc-dependent regulation are potentially invaluable for anticancer therapy. Because c-Myc does not have enzymatic activity or targetable pockets, researchers have attempted to obtain small molecules that inhibit c-Myc cofactors, activate c-Myc repressors, or target epigenetic modifications to regulate the chromatin of c-Myc-addicted cancer without any clinical success. In this study, we screened for c-Myc inhibitors using a cell-dependent assay system in which the expression of c-Myc and its transcriptional activity can be inferred from monomeric Keima and enhanced GFP fluorescence, respectively. We identified one mitochondrial inhibitor, antimycin A, as a hit compound. The compound enhanced the c-Myc phosphorylation of threonine-58, consequently increasing the proteasome-mediated c-Myc degradation. The mechanistic analysis of antimycin A revealed that it enhanced the degradation of c-Myc protein through the activation of glycogen synthetic kinase 3 by reactive oxygen species (ROS) from damaged mitochondria. Furthermore, we found that the inhibition of cell growth by antimycin A was caused by both ROS-dependent and ROS-independent pathways. Interestingly, ROS-dependent growth inhibition occurred only in the presence of c-Myc, which may reflect the representative features of cancer cells. Consistently, the antimycin A sensitivity of cells was correlated to the endogenous c-Myc levels in various cancer cells. Overall, our study provides an effective strategy for identifying c-Myc inhibitors and proposes a novel concept for utilizing ROS inducers for cancer therapy.

The *c-Myc* gene is one of the important members of the Myc gene family (*c*-, *N*-, and *L-Myc*) and encodes a set of transcription factors that feature prominently in cancer (1, 2). The c-Myc protein is a nuclear transcription factor that binds to specific regulatory sequences and forms a transcriptional complex that regulates the transcriptional initiation of target genes (1, 2). c-Myc regulates cell growth and tissue development and plays an important role in numerous cellular processes, such as proliferation, differentiation, and programmed cell death (3, 4, 5). The c-Myc protein expressed at normal levels can participate in cell cycle regulation (6, 7, 8). Various studies have shown that c-Myc deregulation is a hallmark of over 70% of human cancers (9, 10, 11). Furthermore, as critical regulators of proliferation and cell growth, the c-Myc and MAX proteins form a heterodimer and induce the transcription of genes important for regulating cell growth (12). Although it has been established that c-Myc inhibitors are effective in cancer therapy, no effective inhibitor for clinical use has been identified yet.

Elevated levels of c-Myc enhance the accumulation of c-Myc-MAX heterodimers, leading to transcriptional amplification that causes various types of cancer. Thus, studying the molecular mechanisms regulating c-Myc levels can provide new insights into tumor treatment. The ubiquitin–proteasome pathway is an important regulatory mechanism of c-Myc in cells and is one of the major mechanisms responsible for controlling c-Myc levels (13). Two N-terminal phosphorylation sites in c-Myc, threonine 58 (T58) and serine 62 (S62), are critical for determining the stability of c-Myc (14). Phosphorylation of S62 is required for Ras-induced stabilization of c-Myc, which is likely mediated through the action of extracellular signal–regulated kinase (ERK) (15). Conversely, phosphorylation of T58, mediated by glycogen synthetic kinase 3α/β (GSK3α/β) but dependent on prior phosphorylation of S62, is associated with c-Myc degradation (16, 17).

Reactive oxygen species (ROS) are broadly defined as oxygen-containing reactive species, such as hydrogen peroxide, superoxide (O_2_^−^), and the hydroxyl radical (·OH). ROS regulate many cellular functions, such as proliferation, differentiation, and cell death (18), and are involved in several pathological processes, such as cardiovascular diseases, diabetes, neurodegenerative diseases, and cancer (19). Since elevated ROS levels are common among many types of cancer, upregulation of the cellular redox regulation systems has attracted attention as a potential anticancer strategy (20, 21). Indeed, some ROS-inducing compounds are selectively toxic to oncogenically transformed cells than to normal cells (22, 23, 24). However, the molecular mechanisms of ROS regulation systems in cancer cells are not fully understood yet.

We established a novel screening system for c-Myc inhibitors and examined the mechanism of action of the identified c-Myc inhibitor. This study provides a practical strategy for identifying c-Myc inhibitors. In addition, we propose a novel concept for utilizing ROS inducers for cancer therapy.

## Results

### Development and validation of a screening system to identify c-Myc transcription activity inhibitors

**Figure 1:**
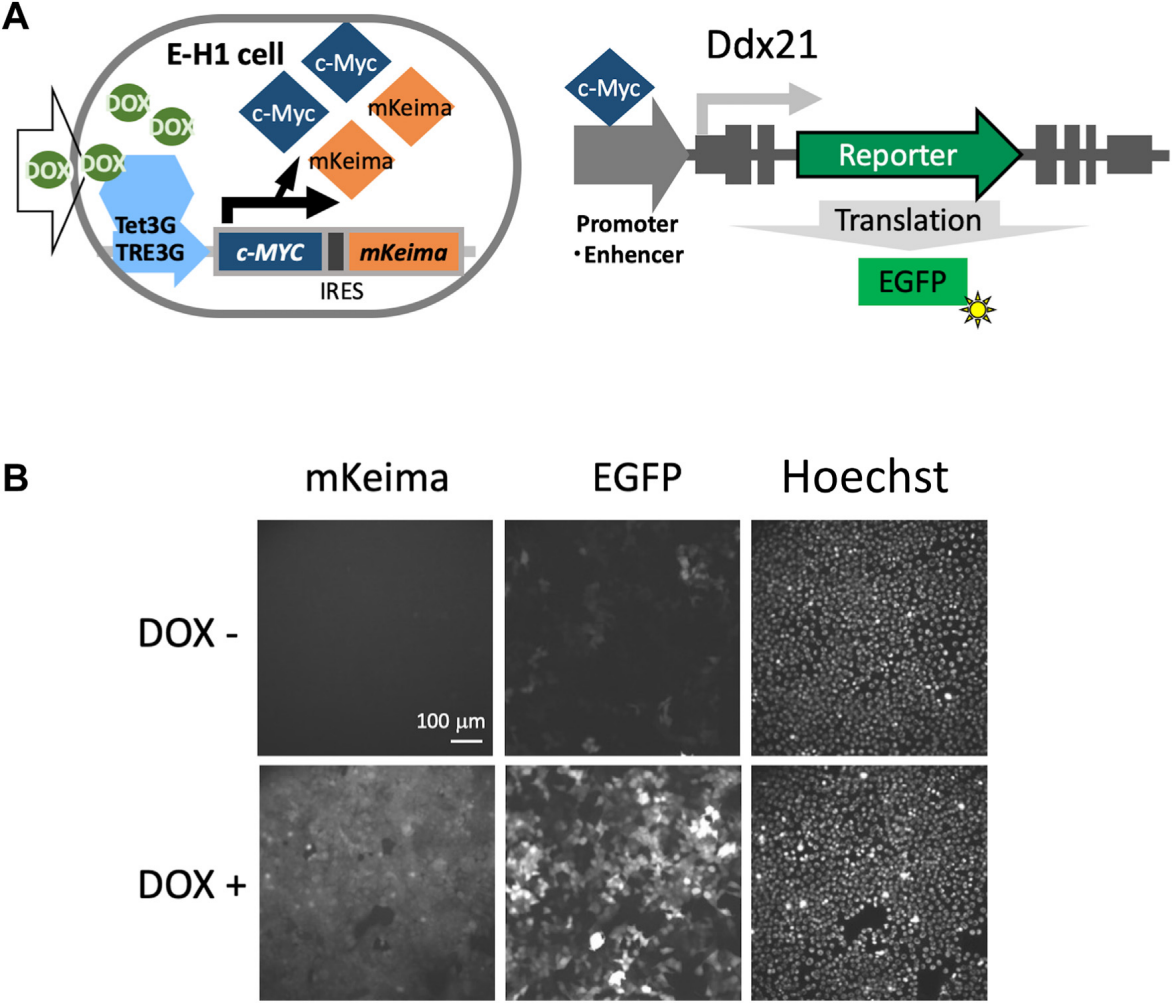
**Development of the screening system.***A*, schematic representation of the modified cell lines. *Left*, c-Myc-IRES-mKeima under the control of the tetracycline-regulated promoter (TRE3G) were inserted into the genome of NMuMG cells so that c-Myc expression by adding doxycycline (DOX) could be monitored by mKeima fluorescent protein. *Right*, in E-H1 cells, the EGFP reporter construct was found to be inserted at the Ddx21 locus (25) and expresses under the control of the Ddx21 promoter activity, which is known to be upregulated by c-Myc, so that the expression of EGFP could be used to monitor c-Myc-regulated gene expression. *B*, E-H1 cells were fixed 24 h after adding DOX (+DOX; 100 ng/ml). The fluorescence of mKeima, EGFP, and Hoechst was analyzed using INCA. EGFP, enhanced GFP; INCA, IN Cell Analyzer 2000; mKeima, monomeric Keima.

A high-throughput screening system was developed to efficiently screen c-Myc inhibitors. E-H1 cells (25), in which c-Myc is expressed under the Tet-ON system together with the fluorescent protein monomeric Keima (mKeima), were used (Fig. 1*A*). Since the ORF of mKeima was inserted after the c-Myc ORF following the internal ribosome entry site, c-Myc expression could be monitored *via* mKeima fluorescence. In the E-H1 cell line, c-Myc transcription activity can be measured *via* enhanced GFP (EGFP), which is inserted into the promoter of DEAD (Asp-Glu-Ala-Asp) box helicase 21 (Ddx21) (25), which is one of the intrinsic transcriptional targets of c-Myc (26) (Fig. 1*A*). To exclude compounds that inhibit transcription generally, a reporter cell line that responds to the expression of an unrelated transcription factor (HNF1B) instead of c-Myc was used as a negative control (10-A6 cells). The expression of EGFP and mKeima and the number of nuclei determined with Hoechst dye staining were analyzed using IN Cell Analyzer 2000 (INCA; GE Healthcare) (Fig. 1*B*).

**Figure 2:**
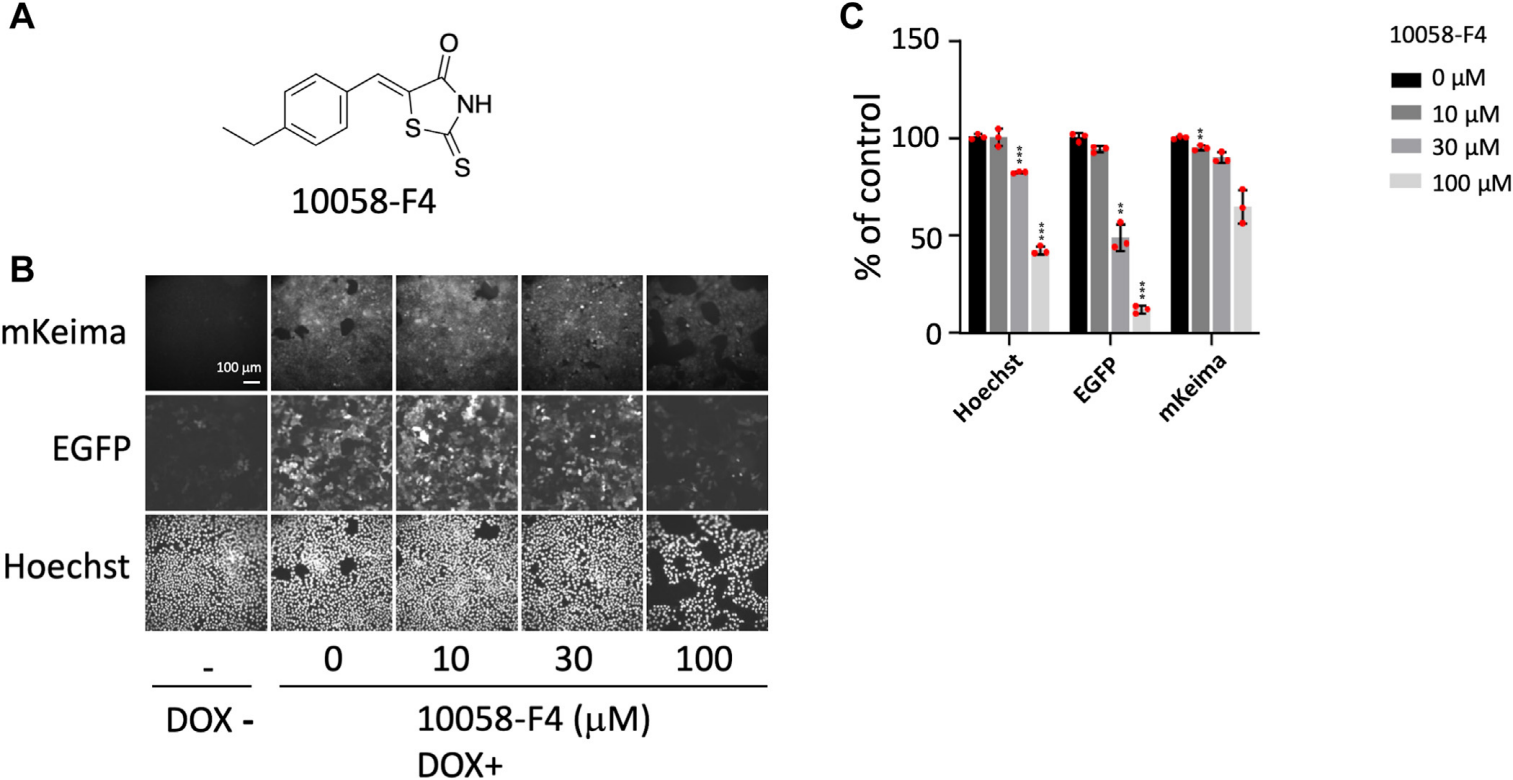
**Validation of the c-Myc inhibitor screening system using the known inhibitor 10058-F4.***A*, the structure of 10058-F4. *B*, E-H1 cells were fixed 24 h after adding doxycycline (DOX; 100 ng/ml) and 10058-F4 (0, 10, 30, and 100 μM) (n = 2). A representative result of two independent experiments is shown. The expression of mKeima and EGFP was analyzed using INCA. Nine cell images were taken from each well and analyzed. Representative images from them are shown. *C*, quantification of EGFP, mKeima, and Hoechst fluorescence in (*B*). The relative values (% of control [DOX+, without 10058-F4]) are shown. Results are shown as mean ± SD (n = 3) (difference from 0 μM; *t* test, ∗∗*p* < 0.01, ∗∗∗*p* < 0.001). The *red dots* indicate the individual measurements. A representative result from two independent experiments is shown. EGFP, enhanced GFP; INCA, IN Cell Analyzer 2000; mKeima, monomeric Keima.

To determine the reliability of this system, the inhibitory effect of 10058-F4, which is a c-Myc inhibitor that blocks the c-Myc-MAX interaction (IC_50_ = 49 μM in HL-60 cells), on c-Myc activity (27, 28) was investigated (Fig. 2*A*). Compound 10058-F4 inhibited the expression of EGFP in a concentration-dependent manner but had only a slight effect on the expression of mKeima (Fig. 2, *B* and *C*), which demonstrated that this assay is well suited for small-molecule inhibitor screening.

### Antimycin A as a c-Myc transcriptional inhibitor

**Figure 3:**
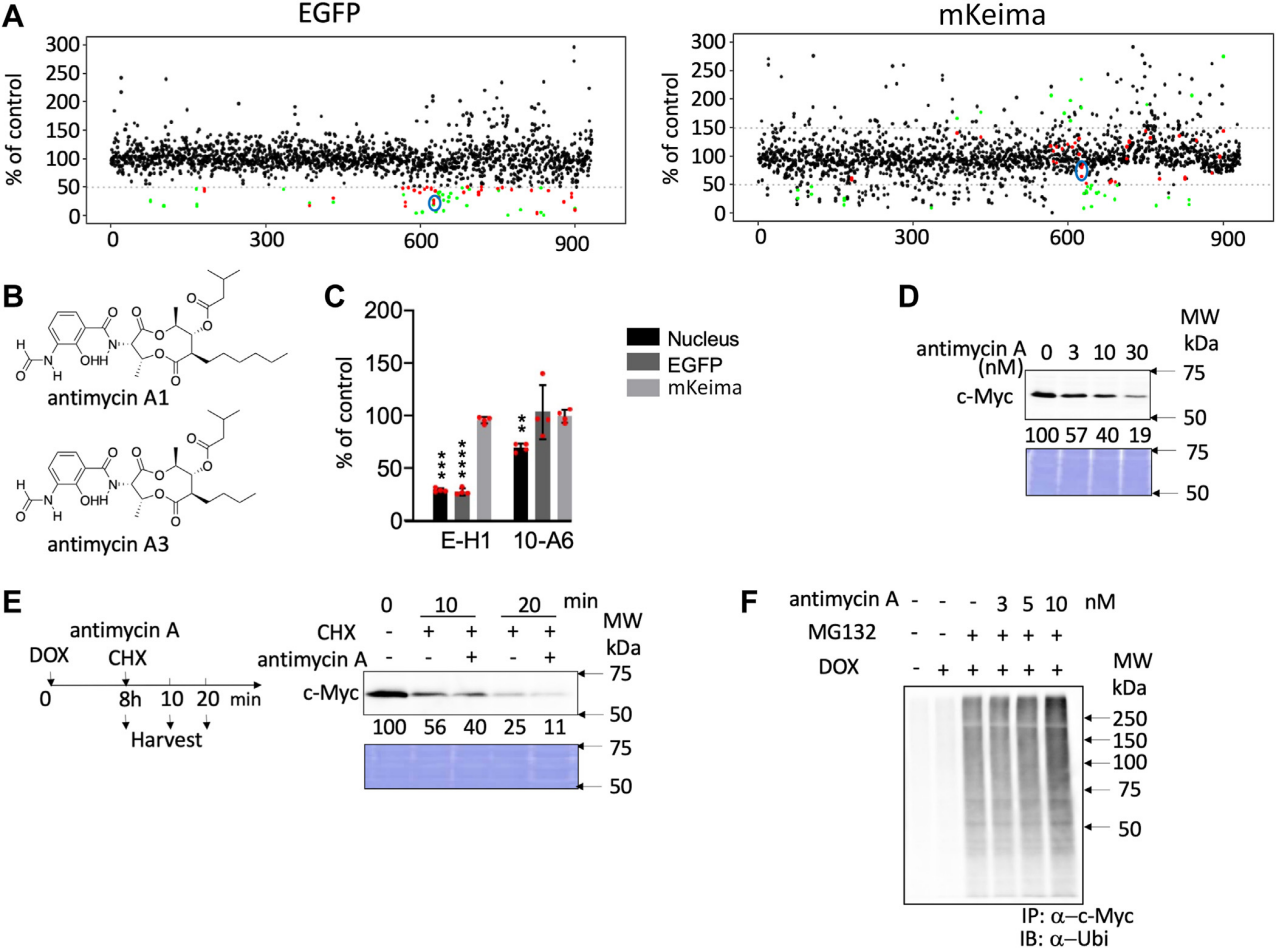
**Identification of antimycin A as c-Myc inhibitor and analyses ofthe mechanism of inhibition.***A*, results of initial screening of 987 compounds (n = 2). E-H1 cells were fixed 24 h after adding doxycycline (DOX) and compounds. The expression of EGFP (*left*) and mKeima (*right*) was analyzed using INCA, which were standardized, and is represented as a percentage value of control (with DOX, without compound; *i.e.*, 100% means no inhibition). When the compounds reduced EGFP fluorescence to less than 50% of the control, the *dots* were colored in *red* or *green* in the panel using the criteria as follows. When the mKeima fluorescence (*right panel*) in the assay was not affected (between 50 and 150% of control), the *dots* were colored in *red* in both panels, whereas the mKeima was affected (out of the range above), the *dots* were colored in *green*. When the mKeima value was more than 300 in at least one assay, the compounds (41 compounds) were removed from the plot. *Red dots* in *blue circle* indicated ISN-055 (antimycin A). The robustness of the screening was confirmed by the Z′ factors of each plate (Fig. S1). *B*, structure of antimycin A1 and A3. *C*, the c-Myc-specific transcriptional inhibition by antimycin A. E-H1 and 10-A6 cells were fixed for 24 h after adding 100 ng/ml DOX and antimycin A (10 nM). The relative values (% of control [DOX+, without antimycin A]) as mean ± SD are shown (n = 4). The *red dots* indicate the individual measurements; ∗∗, ∗∗∗, and ∗∗∗∗ indicate significant difference from control (100%) in *t* test (*p* < 0.01, *p* < 0.001, and *p* < 0.0001, respectively). A combined result of two independent experiments is shown; a similar result is also obtained in a separate experiment. *D*, dose-dependent effects of antimycin A on c-Myc levels in E-H1 cells. E-H1 cells were harvested and lysed after treatment with 100 ng/ml DOX and antimycin A for 24 h, and c-Myc levels were analyzed by immunoblotting. CBB staining is also shown as a loading control. Quantification of protein levels was performed using ImageJ and is shown as the percentage of cells not treated with antimycin A. The result is selected from two independent experiments. *E*, c-Myc degradation by antimycin A was analyzed using cycloheximide (CHX) chase. E-H1 cells were treated with 10 nM antimycin A with 40 μg/ml CHX 8 h after 100 ng/ml DOX addition and harvested at 0, 10, and 20 min thereafter. c-Myc levels were analyzed by immunoblotting. CBB staining is also shown as a loading control. Quantification of protein levels was performed using ImageJ and is shown as the percentage of cells without antimycin A and CHX at 0 (8 h). The result is selected from two independent experiments. *F*, increased ubiquitination of c-Myc by antimycin A in E-H1 cells. Cells were harvested and lysed after treatment with antimycin A with or without MG132 (6 μM) for 6 h. c-Myc proteins of each sample were harvested from 300 μg lysates using anti-c-Myc antibody-conjugated beads, and their ubiquitination was analyzed by immunoblotting. The result is selected from two independent experiments. CBB, Coomassie brilliant blue; EGFP, enhanced GFP; INCA, IN Cell Analyzer 2000; mKeima, monomeric Keima; MW, molecular weight.

Approximately 1000 compounds from the RIKEN Natural Products Depository (NPDepo) were examined (Figs. 3*A* and S1), and ISN-055 showed strong activity even at low concentrations. ISN-055 has already been identified as a mixture of antimycin A1 and A3 (see “Compounds” in the Experimental procedures section), and we used ISN-055 as antimycin A for further analyses (Fig. 3*B*).

To confirm the specific effect of antimycin A on c-Myc, we examined the effect of antimycin A on HNF1B in 10-A6 cells and on c-Myc in E-H1 cells. Antimycin A reduced the fluorescence of EGFP to less than 50% of control levels only in E-H1 cells but not in 10-A6 cells, indicating that antimycin A did not generally inhibit transcription. (Fig. 3*C*). In this context, there is a possibility that 10-A6 cells may have low antimycin A uptake. However, antimycin A inhibited 10-A6 cell growth at a lower level, possibly because it lacks c-Myc (see later). Therefore, we assume that the uptake of the antimycin A in 10-A6 cells is not low.

### Antimycin A treatment reduces c-Myc protein levels

Although the mKeima level, which reflects the expression of c-Myc induced by doxycycline (DOX), was not affected by antimycin A, we examined the expression of c-Myc after treatment with antimycin A. We noticed that antimycin A led to a reduction in c-Myc protein levels 1 day after treatment in a concentration-dependent manner (Fig. 3*D*). There are two possible explanations for this observation. The reduction in c-Myc expression might be caused by either synthesis inhibition or the acceleration of degradation. To corroborate this finding, we used the cycloheximide chase assay to block the synthesis of c-Myc and found that antimycin A enhanced c-Myc protein degradation (Fig. 3*E*). We then examined whether this enhanced c-Myc degradation was caused by the ubiquitin–proteasome system. To inhibit proteasome-dependent degradation, the proteasome inhibitor MG132 was added to E-H1 cells together with antimycin A for 6 h after c-Myc induction by DOX. c-Myc protein was immunoprecipitated from an equal amount of cell lysates, and the ubiquitination of c-Myc was examined by immunoblotting with an antiubiquitin antibody. After treatment with antimycin A, ubiquitinated c-Myc levels increased in a concentration-dependent manner (Fig. 3*F*). These results indicate that antimycin A enhances ubiquitin-dependent c-Myc degradation.

### c-Myc degradation by antimycin A treatment was abolished in the presence of alsterpaullone and CT99021

**Figure 4:**
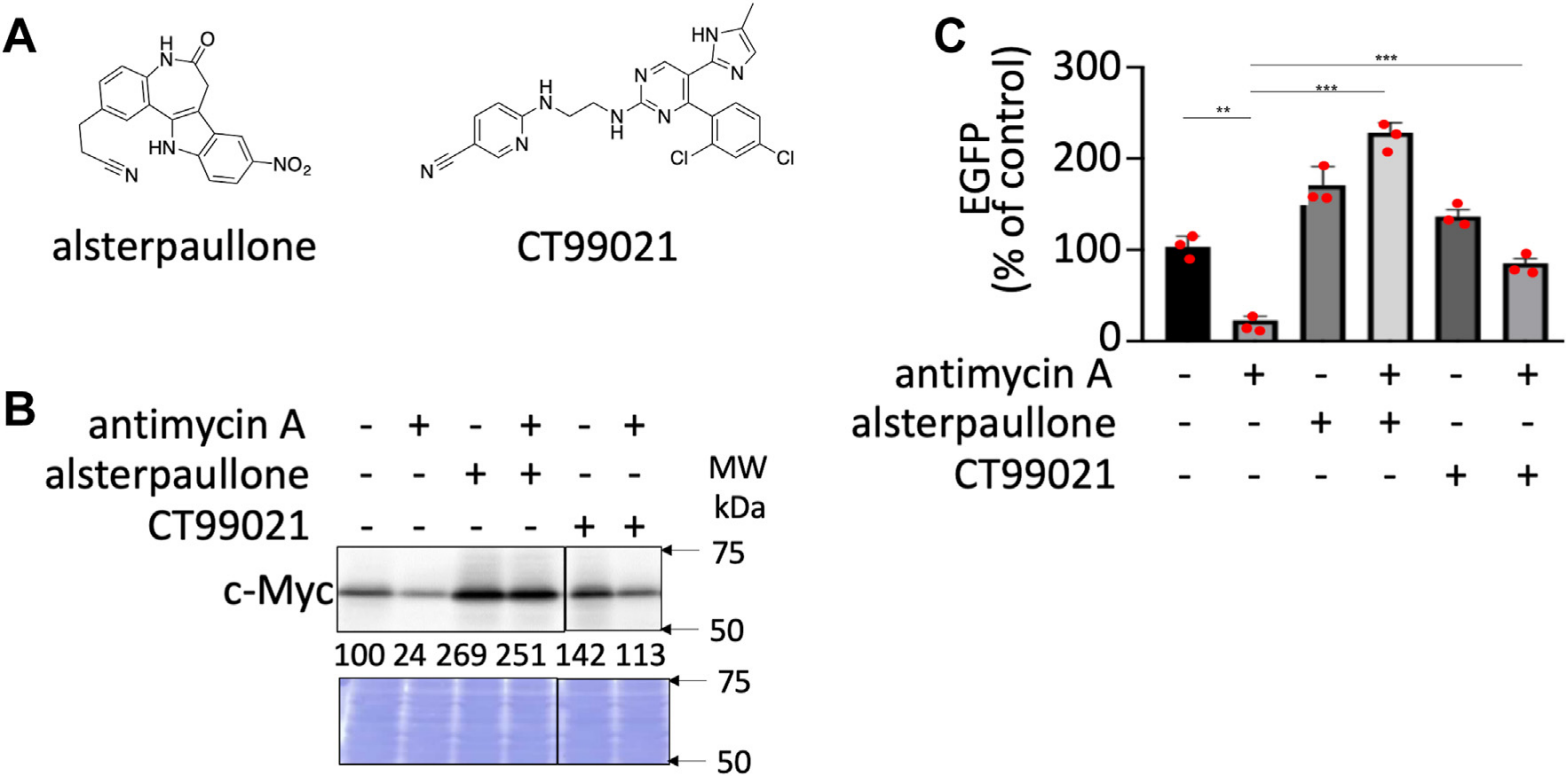
**Mechanism of c-Myc degradation by antimycin A in E-H1 cells.***A*, structures of alsterpaullone (CDK and GSK3α/β inhibitor) and CT99021 (GSK3α/β inhibitor). *B*, c-Myc protein reduction by antimycin A (10 nM) was inhibited by alsterpaullone (1 μM) and CT99021 (10 μM). Compounds were added to 10 nM antimycin A and 100 ng/ml doxycycline for 24 h, harvested, and lysed. c-Myc levels were analyzed by immunoblotting. CBB staining is also shown as a loading control. Quantification of c-Myc levels was performed using ImageJ and shown as a percentage of cells without antimycin A and compounds. A representative result of two independent experiments is shown. *C*, the effect of alsterpaullone (1 μM) and CT99021 (10 μM) on the effect of antimycin A (10 nM) on c-Myc activity was monitored by EGFP levels using INCA. EGFP levels are shown as the percentage of cells without antimycin A and compounds. Results are shown as mean ± SD (n = 3) and are representative of two independent experiments. (∗∗*p* < 0.01, ∗∗∗*p* < 0.001 by *t* test). The *bars* indicate SD, whereas *red dots* indicate the individual measurements. CBB, Coomassie brilliant blue; CDK, cyclin-dependent kinase; EGFP, enhanced GFP; GSK3α/β, glycogen synthetic kinase 3 α/β; INCA, IN Cell Analyzer 2000; MW, molecular weight.

The stability of c-Myc is known to be regulated by several mechanisms, among which the ordered phosphorylation cascade, wherein c-Myc phosphorylation on S62 by protein kinases, such as ERK, cyclin-dependent kinase, and c-Jun N-terminal kinase, primes subsequent phosphorylation on T58 (pT58) by GSK3α/β, is considered one of the main mechanisms (15, 16, 29, 30). When the pT58 of c-Myc is recognized by E3 ubiquitin ligase, c-Myc is ubiquitinated and degraded by 26S proteasome (13, 14, 31). Thus, we investigated whether antimycin A affects c-Myc protein stability through this mechanism. To test this hypothesis, we used two compounds: alsterpaullone, which inhibits GSK3α/β and is shown to inhibit GSK3β by inducing the phosphorylation of S9 of GSK3β (32), and CT99021, a GSK3α/β inhibitor (33) (Fig. 4*A*). Antimycin A was added with/without these two compounds; results showed that the reduction in c-Myc protein levels because of antimycin A was rescued by alsterpaullone and CT99021 (Fig. 4*B*). Consistent with the recovery of c-Myc protein levels, the reduction in EGFP fluorescence because of antimycin A was also reversed by alsterpaullone and CT99021 in a cell-based assay using INCA (Fig. 4*C*). These results indicated that GSK3α/β plays a role in enhanced c-Myc degradation and that antimycin A promotes the phosphorylation cascade to enhance c-Myc degradation.

### Endogenous c-Myc degradation by antimycin A in cancer cells

**Figure 5:**
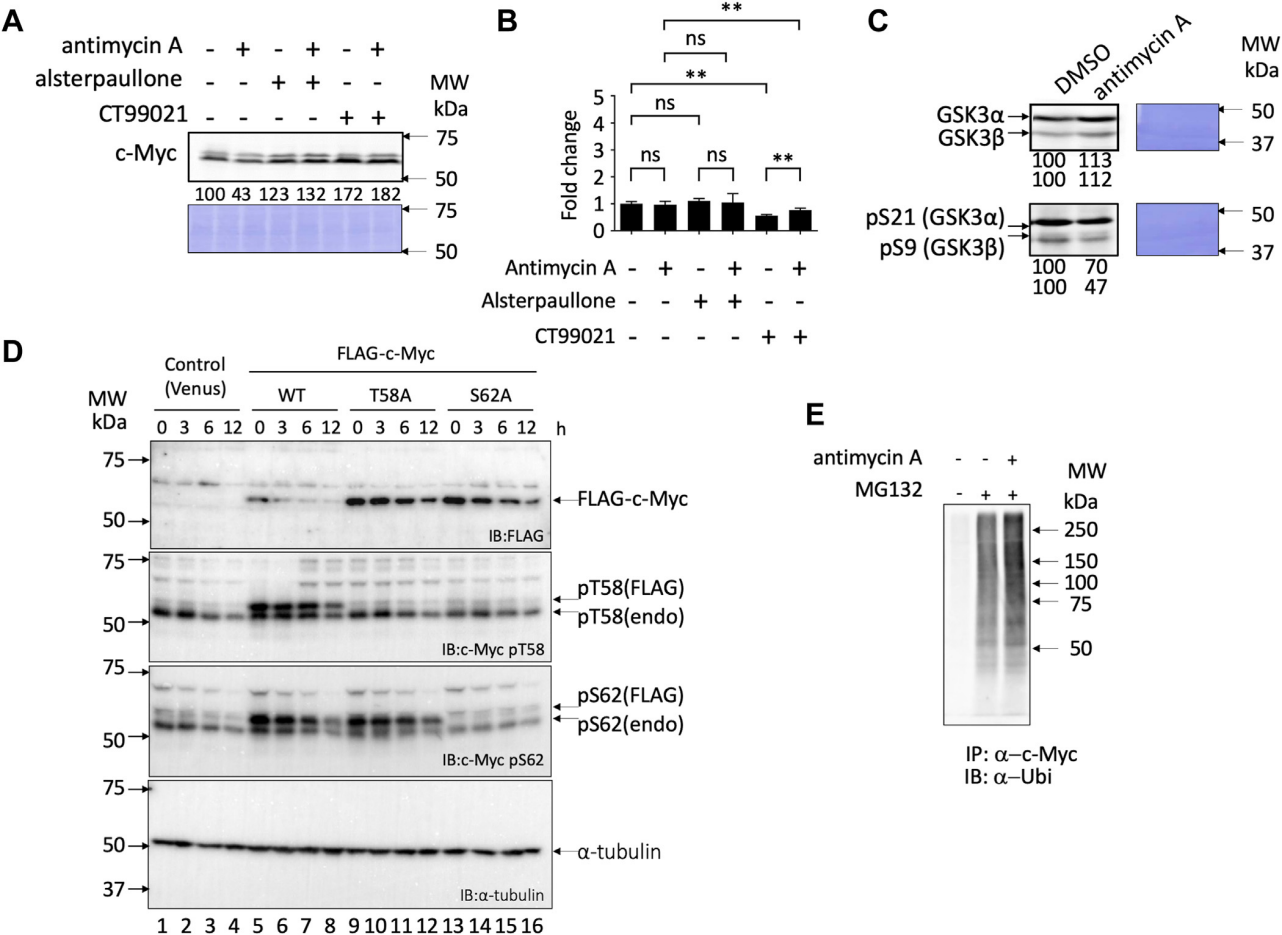
**Mechanism of c-Myc protein degradation by antimycin A in HCT116 cells.***A*, c-Myc protein reduction by antimycin A (10 nM) was inhibited by alsterpaullone (1 μM) and CT99021 (10 μM) in the HCT116 cells. Compounds were added with or without 10 nM antimycin A for 24 h, harvested, and lysed, and c-Myc levels were analyzed by immunoblotting. CBB staining is also shown as a loading control. Quantification of protein levels was performed using ImageJ and shown as the percentage of cells without antimycin A and compounds. A representative result of two independent experiments is shown. *B*, mRNA levels of c-Myc after the treatment with the compounds were analyzed by real-time PCR analyses (n = 3). Endogenous mRNA levels of GAPDH were used as a relative internal control. Results are shown as mean ± SD of three experiments (three measurements each). ∗∗*p* < 0.01 by *t* test; ns, not significant. *C*, effect of antimycin A on the levels of GSK3α/β and their phosphorylation in HCT116 cells. Cells were harvested and lysed after treatment with antimycin A for 24 h and analyzed by immunoblotting. CBB staining is also shown as a loading control. Quantification of protein levels was performed using ImageJ and is shown as the percentage of cells without antimycin A. A representative result of two independent experiments is shown. *D*, time-dependent effect of antimycin A on the level and phosphorylation of wildtype and T58 and S62 mutants of c-Myc. FLAG-tagged c-Myc (WT, T58A, and S62A) was introduced by retrovirus vector in HCT116 cells. After the addition of antimycin A (10 nM), cells were harvested at the time indicated, and the levels of c-Myc and its phosphorylation at T58 and S62 were examined by immunoblotting using anti-FLAG, anti-pT58, and anti-pS62 antibodies, respectively. Anti-α-tubulin blot as a loading control is also shown. A representative result of two independent experiments is shown. *E*, increased ubiquitination of c-Myc by antimycin A (10 nM) in HCT116 cells. Cells were harvested and lysed after treatment with antimycin A with or without MG132 (6 μM) for 6 h. c-Myc proteins of each sample were harvested from 300 μg lysates using anti-c-Myc antibody-conjugated beads, and their ubiquitination was analyzed by immunoblotting. A representative result of two independent experiments is shown. CBB, Coomassie brilliant blue; GSK3α/β, glycogen synthetic kinase 3 α/β; MW, molecular weight; ns, not significant.

Next, we examined whether antimycin A induced degradation of endogenous c-Myc in cancer cells. HCT116, HeLa, MIA PaCa-2, A549, PANC-1, and HL-60 cancer cells were treated with antimycin A for 24 h, and the levels of endogenous c-Myc protein were examined (Fig. S2). The reduction in c-Myc levels because of antimycin A was observed in all these cells, but it was most apparent in HCT116 cells. To investigate whether antimycin A also affects endogenous c-Myc protein stability through the phosphorylation-dependent ubiquitin–proteasome system, HCT116 cells were treated with antimycin A with or without alsterpaullone and CT99021. Consistent with the results in E-H1 cells, the reduction in c-Myc because of antimycin A was abolished in the presence of alsterpaullone and CT99021 (Fig. 5*A*), indicating that GSK3α/β acted on c-Myc protein reduction. Therefore, we also investigated whether antimycin A or the GSK3 inhibitors affects the mRNA level of c-Myc using RT–quantitative PCR technique (Fig. 5*B*). However, the mRNA level of c-Myc was not decreased by antimycin A treatment and was not increased by the GSK3 inhibitor treatment, showing that the change of c-Myc protein level is caused post-transcriptionally. GSK3α/β phosphorylates the T58 of c-Myc; thus, we speculated that GSK3α/β is activated by antimycin A. Evidently, treatment with antimycin A decreased the levels of the inhibitory phosphorylation at S21 and S9 of GSK3α and β, respectively (Fig. 5*C*), which activated GSK3α/β to promote c-Myc reduction. To confirm c-Myc was degraded through the T58 phosphorylation, we introduced FLAG-tagged c-Myc or its T58A mutant into HCT116 cells and examined the effects of antimycin A on their time-dependent stability by using anti-FLAG antibody immunoblotting (Fig. 5*D*, FLAG *panel*). The introduced wildtype c-Myc was rapidly degraded after the addition of antimycin A (lanes 5–8), whereas the degradation of T58A mutant was significantly delayed (lanes 9–12). The T58 phosphorylation of c-Myc is known to be primed by the phosphorylation of S62 (15, 16, 29, 30). The degradation of S62A mutated c-Myc was also delayed (lanes 13–16). The absence of T58 phosphorylation in S62A mutant of c-Myc shown by the pT58-specific antibody blotting clearly indicates the S62-dependent phosphorylation of T58 (lanes 13–16, pT58 *panel*). These results indicate that the degradation of c-Myc by antimycin A was induced through the T58 phosphorylation. Treatment with antimycin A elevated c-Myc ubiquitination in HCT116 cells, similarly in E-H1 cells (Fig. 5*E*).

### c-Myc-dependent cancer cell viability is inhibited by antimycin A through ROS induction

**Figure 6:**
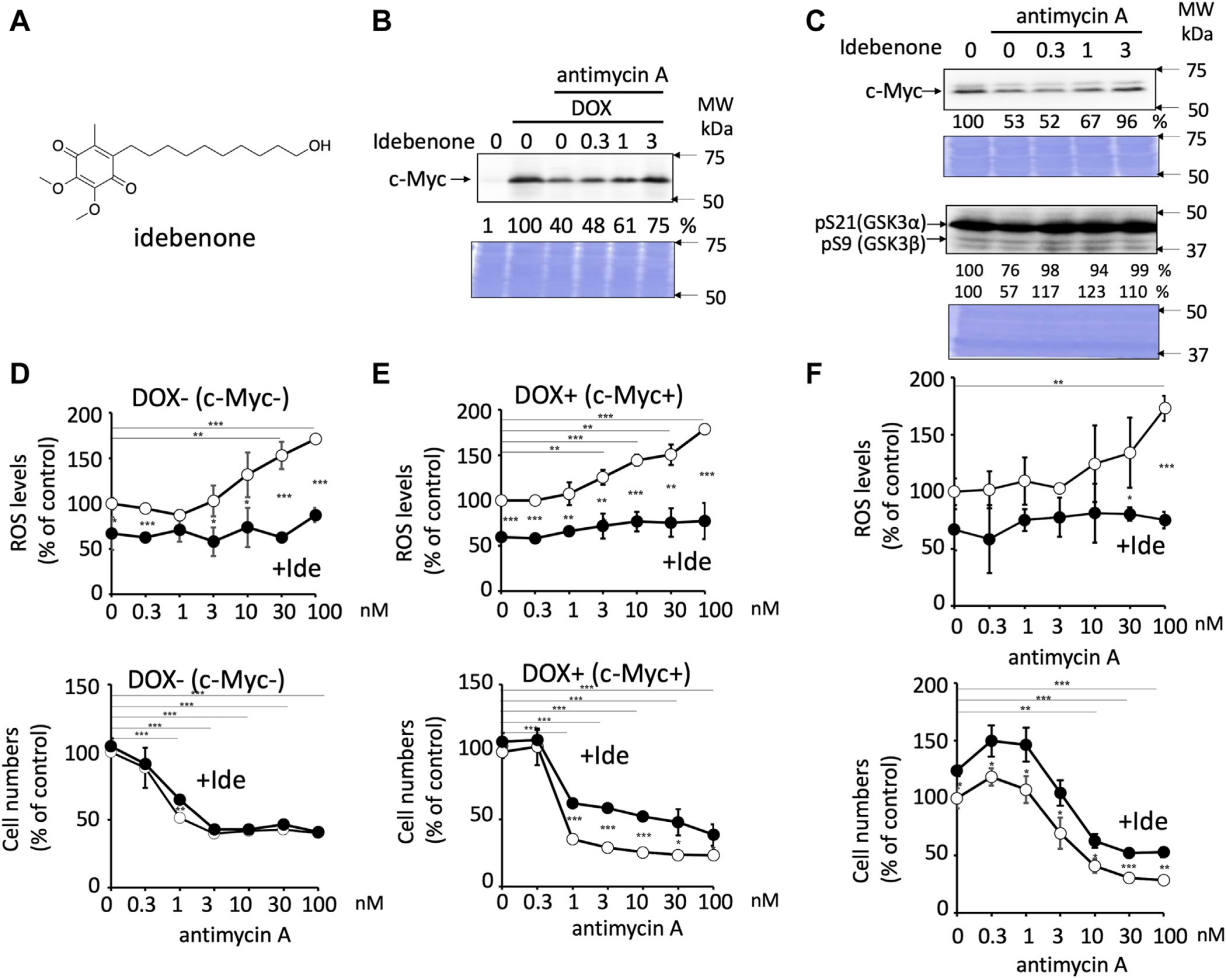
**ROS produced by antimycin A inhibits cell growth only in the presence of c-Myc.***A*, the structure of idebenone. *B*, reduction in c-Myc protein levels because of antimycin A was inhibited by idebenone in E-H1 cells. Idebenone at the indicated concentrations was added with antimycin A (10 nM) and DOX (100 ng/ml) for 24 h, and the cells were harvested and lysed. c-Myc protein levels were analyzed by immunoblotting, quantitated by ImageJ, and shown in the percent of that of control cells (with DOX, without antimycin A). CBB staining is also shown as a loading control. A representative result of two independent experiments is shown. *C*, reduced c-Myc protein levels and pS21/pS9 of GSK3α/β levels by antimycin A were inhibited by idebenone in HCT116 cells. Idebenone at the indicated concentrations was added with antimycin A (10 nM) for 24 h; cells were harvested and lysed. c-Myc protein levels and pS21/9 of GSK3α/β levels were analyzed by immunoblotting, quantitated by ImageJ, and shown as the percentage of that of control (without compounds) cells. CBB staining is also shown as a loading control. *D*, E-H1 cells were cultured in the absence of DOX, antimycin A at the indicated concentrations was added with (+Ide, *closed circles*; 1 μM) or without (*open circles*) idebenone, and cultured for 1 h. Next, the cells were fixed, and mitochondrial ROS levels (*top*) were measured. Or cultured for 48 h, fixed, and cell growth was measured by counting the number of nuclei after Hoechst staining (*bottom*). Results are shown as mean ± SD (n = 3), and a representative result of two independent experiments. ANOVA test, ∗*p* < 0.05, ∗∗*p* < 0.01, and ∗∗∗*p* < 0.001. *E*, E-H1 cells were cultured in the presence of DOX (100 ng/ml), treated with antimycin A and idebenone as in *D*, and the mitochondrial ROS levels (*top*) and cell numbers (*bottom*) were measured as in *D*. *F*, HCT116 cells were treated with antimycin A and idebenone as in *D*, and the mitochondrial ROS levels (*top*) and cell numbers (*bottom*) were measured as in *D*. CBB, Coomassie brilliant blue; DOX, doxycycline; GSK3α/β, glycogen synthetic kinase 3 α/β; MW, molecular weight; ROS, reactive oxygen species.

Since antimycin A is an inhibitor of complex III of mitochondria, we speculated that oxidative stress after mitochondrial damage caused by antimycin A might produce ROS, which might be responsible for activating GSK3 to induce c-Myc protein degradation. To examine this possibility, we used idebenone, a potent antioxidant that can effectively inhibit the effect of ROS (Fig. 6*A*). As expected, when idebenone was added together with antimycin A to E-H1 and HCT116 cells, idebenone abolished the reduction of c-Myc protein levels by antimycin A in a dose-dependent manner (Fig. 6, *B* and *C*, *upper panel*). The reduction in pS21 and pS9 levels of GSK3α and β by antimycin A, respectively, was also abolished in the presence of idebenone (Fig. 6*C*, *lower panel*). These results indicated that ROS production induced by damaged mitochondria activated GSK3α and β and reduced c-Myc protein levels.

We confirmed that antimycin A in E-H1 cells increased mitochondrial ROS levels in a dose-dependent manner both in the absence and presence of DOX (Fig. 6, *D* and *E*, *upper panel*). The increase in ROS production was inhibited on adding idebenone (Fig. 6, *D* and *E*, *upper panel*). We examined the effect of ROS produced by antimycin A on cell growth (Fig. 6, *D* and *E*, *lower panel*). In the absence of DOX, antimycin A inhibited cell growth, and idebenone did not have any effect (Fig. 6*D*, pT58, *lower panel*). Interestingly, however, in the presence of DOX (*i.e.*, c-Myc expression), antimycin A also inhibited cell growth, but idebenone could partially abolish it (Fig. 6*E*, *lower panel*). Thus, these results indicate that ROS-dependent growth inhibition occurs only in the presence of c-Myc. Therefore, c-Myc expression may add the features of cancer cells to normal cells such as NMuMG cells. Thus, the difference in ROS-dependent growth inhibition between the absence and presence of c-Myc may reflect the difference between normal and cancer cells. In HCT116 cancer cells, cell growth inhibition because of antimycin A was consistently partially abolished by idebenone (Fig. 6*F*).

**Figure 7:**
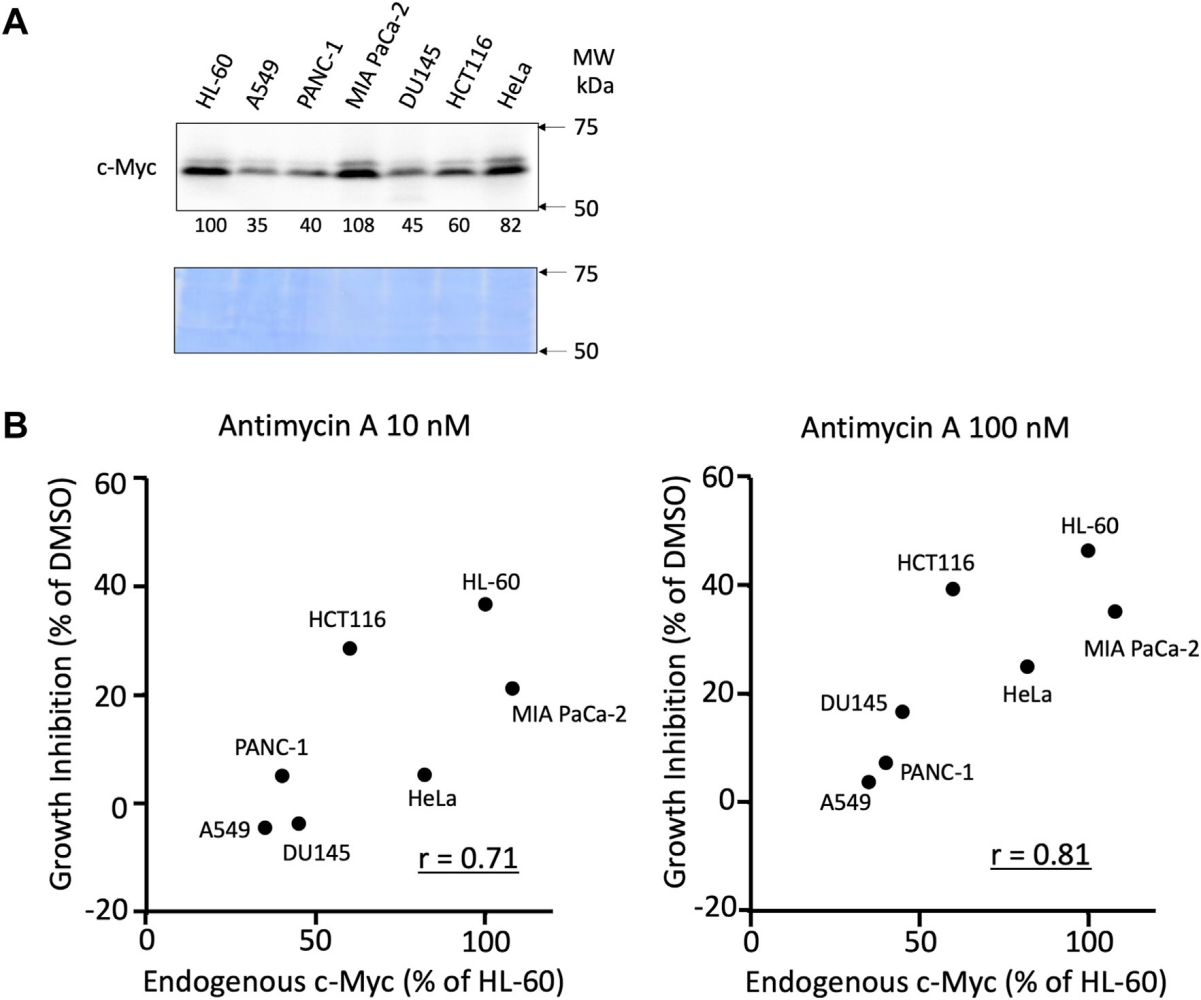
**Growth inhibition of cancer cells by antimycin A correlates with the endogenous levels of c-Myc protein.***A*, endogenous levels of c-Myc in seven human cancer cell lines indicated in the figure were examined by immunoblotting of whole cell lysates, quantitated by ImageJ, and shown as the percentage of that of HL-60 cells. CBB staining is also shown as a loading control. A representative result of two independent experiments is shown. *B*, correlation between endogenous c-Myc level and growth inhibition by antimycin A at 10 nM (*left*) and 100 nM (*right*) for 24 h was analyzed. Correlation coefficient was calculated and shown in the panels. A representative result of two independent experiments is shown. CBB, Coomassie brilliant blue; MW, molecular weight.

Antimycin A induces c-Myc degradation at different rates in various cancer cells (Fig. S2). As hypothesized previously, if antimycin A-dependent c-Myc degradation inhibits cell growth in cancer cells, there should be a correlation between the c-Myc degradation and growth inhibition by antimycin A. As predicted, we found a positive relationship between c-Myc degradation and the growth inhibition caused by antimycin A at 100 nM. However, this relationship was not clear at 10 nM antimycin A, possibly because of the low reduction rate of c-Myc at 10 nM (Fig. S3). Although the parameters responsible for the difference in c-Myc degradation rate among cell lines are still unknown, the previous result indicates the role of c-Myc degradation on the growth inhibition by antimycin A. In addition, we observed that cell lines with high level of endogenous c-Myc showed greater sensitivity to antimycin A (Fig. 7). The correlation coefficients between endogenous c-Myc level and growth inhibition by 10 and 100 nM antimycin A were 0.71 and 0.81, respectively. These results indicate the involvement of c-Myc in the antimycin A sensitivity.

## Discussion

c-Myc overexpression causes abnormal proliferation, leading to many types of cancers. Reflecting its critical role, screening for c-Myc inhibitors has been attempted for a long time (12). Because c-Myc does not have enzymatic activity or targetable pockets, it has not been considered a druggable protein. However, research has attempted to obtain small molecules that inhibit c-Myc cofactors or activate c-Myc repressors (12, 34). Targeting epigenetic modifications to regulate the chromatin of c-Myc-addicted cancer is another approach to inhibit c-Myc function (12, 34). In contrast to these previous studies, we used a high-throughput cell-based screening system to identify valuable c-Myc inhibitors that act on cells. In other words, without targeting the specific features of c-Myc, we tried to obtain small molecules that specifically inhibit c-Myc transcriptional activity in cells using a high-sensitivity screening system with E-H1 cells, in which c-Myc expression and transcriptional activity were monitored using mKeima and EGFP, respectively. In negative control 10-A6 cells, the unrelated transcription factor HNF1B was expressed instead of c-Myc, and its expression was monitored using mKeima. In 10-A6 cells, the transcriptional activity of HNF1B was monitored by EGFP. Therefore, if a compound can interrupt the expression of EGFP, but not mKeima, only in E-H1 cells, it may be considered a specific inhibitor of c-Myc transcription activity.

After screening the RIKEN NPDepo libraries, we found that antimycin A seemed to inhibit c-Myc transcriptional activity. Although the coexpressed mKeima protein, which reflects the expression level of c-Myc, was not affected by antimycin A, we found that the expression level of c-Myc was reduced after treatment with antimycin A, indicating that the translation of c-Myc was not affected, but its degradation was accelerated by antimycin A. The ubiquitin–proteasome pathway is one of the major pathways by which c-Myc levels are controlled in cells (13). The phosphorylation of S62 in c-Myc is regulated by the Ras–ERK signaling pathway and is known to be necessary for T58 phosphorylation (29), which is regulated by GSK3 (15). The phosphorylation of T58 promotes c-Myc ubiquitination (15). We found an increased phosphorylated T58 and ubiquitination of c-Myc after adding antimycin A. Moreover, the degradation of c-Myc by antimycin A was significantly delayed in T58A mutant c-Myc. These results indicate that antimycin A induces ubiquitin–proteasome-dependent degradation of c-Myc through T58 phosphorylation by GSK3.

GSK3 is a highly conserved serine/threonine-protein kinase containing two isomers: GSK3α and GSK3β (30). The activity of GSK3α is inhibited by the phosphorylation of S21, whereas GSK3β is inhibited by the phosphorylation of S9 (16, 35). Since GSK3α/β is known to be responsible for increased c-Myc degradation, we speculated that the antimycin A-mediated degradation of c-Myc is caused by GSK3α/β activation. Antimycin A treatment consistently reduced the phosphorylation of these inhibitory phosphorylation sites of GSK3α/β. The recovery from the antimycin A-mediated degradation of c-Myc by the inhibition of GSK3 by alsterpaullone or CT99021 also showed that GSK3 is involved in the antimycin A-induced degradation of c-Myc. In addition to the GSK3 inhibitors used in this study, several other inhibitors showed an increased effect on c-Myc protein levels. A GSK3 inhibitor, named AZD2858, led to an increase of c-Myc basal level (36). Treatment with other inhibitors of GSK3 such as SB415286 and lithium salts also resulted in increased levels of c-Myc in lymphoid cells (37). In SH-SY5Y cells, both alsterpaullone and LiCl could reverse the decrease of c-Myc protein induced by 1-methyl-4-phenylpyridinium ion dose-dependently, and alsterpaullone was more effective than LiCl in the protection of mitochondrial fission caused by 1-methyl-4-phenylpyridinium ion (38). In addition, another GSK3 inhibitor, SB216763, reduced ponatinib-induced cytotoxicity and degradation of c-Myc in leukemia cells (39). These findings indicate that GSK3α/β is responsible for the regulation of c-Myc protein level in multiple situations.

Since antimycin A inhibits complex III in mitochondria, ROS produced by mitochondrial damage may be responsible for the activation of GSK3α/β. ROS-producing compounds are known to induce the activation of GSK3 (40, 41). Consistently, the antioxidant idebenone recovered the reduced c-Myc protein levels and activated GSK3α/β through antimycin A. Thus, antimycin A induces c-Myc degradation through the induction of mitochondrial ROS.

In E-H1 cells without DOX addition, antimycin A inhibited cell growth, whereas idebenone did not recover cell growth. Interestingly, in cells expressing c-Myc, idebenone could partially abolish growth inhibition by antimycin A. These results indicate that the inhibition of cell growth by antimycin A is caused by ROS-independent and -dependent pathways, with only the latter pathway being c-Myc dependent. A ROS-dependent pathway was also observed in HCT116 cells. Our results showed that antimycin A induced ROS production and c-Myc protein degradation, resulting in cell growth inhibition.

In various cancer cells, antimycin A induces c-Myc degradation at different rates (Fig. S2). We found a positive relationship between c-Myc degradation and the growth inhibition by antimycin A at 100 nM (Fig. S3). The positive relationship between c-Myc degradation and the growth inhibition by antimycin A indicates the role of c-Myc degradation on the growth inhibition by antimycin A. In addition, we observed that cell lines with high level of endogenous c-Myc showed greater sensitivity to antimycin A (Fig. 7). These results indicate the involvement of c-Myc for the antimycin A sensitivity.

**Figure 8:**
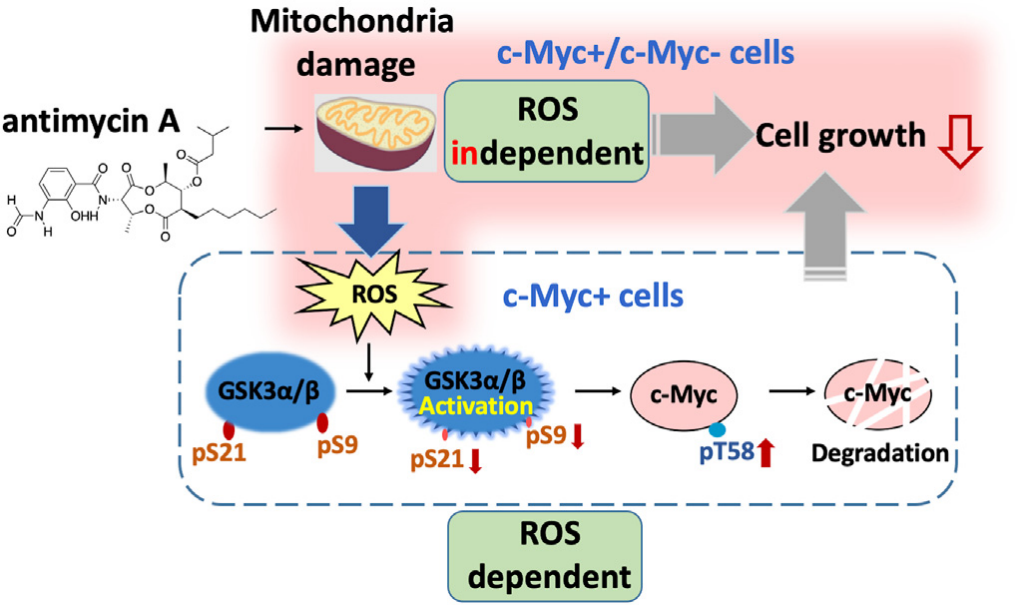
**Antimycin A inhibits cell growth through two pathways.** Antimycin A caused mitochondrial damage, which inhibited cell growth. This inhibition resulted from two pathways; one that does not depend on ROS and occurs in the presence and absence of c-Myc and the other that depends on ROS and occurs only in the presence of c-Myc. ROS are produced from damaged mitochondria and activate GSK3α/β through decreased phosphorylation at S21 and S9, resulting in increased phosphorylation at T58 of c-Myc to induce degradation. GSK3α/β, glycogen synthetic kinase 3 α/β; ROS, reactive oxygen species.

In summary, antimycin A caused mitochondrial damage, which inhibited cell growth. Our results show that this inhibition resulted from two pathways; one that does not depend on ROS and occurs in the presence and absence of c-Myc and an ROS-dependent pathway that occurs only in the presence of c-Myc. ROS are produced from damaged mitochondria and activate GSK3α/β through decreased phosphorylation at S21 and S9, resulting in increased phosphorylation at T58 of c-Myc to induce degradation (Fig. 8).

Different types of tumor cells have been reported to produce elevated levels of ROS compared with their normal counterparts. In other words, the basal level of ROS in cancer cells is higher than that in normal cells, suggesting that cancer cells are more sensitive to further increases in ROS than their normal counterparts. This is a well-accepted mechanism for the use of ROS producers as cancer cell inhibitors (21). Therefore, our finding that ROS inducers inhibit cell growth only in the presence of c-Myc supports the application of ROS producers in cancer treatment.

## Experimental procedures

### Compounds

The test compounds (400 compounds from the new pilot library and 560 natural products and derivatives) were obtained from the RIKEN NPDepo. The compounds were dissolved in dimethyl sulfoxide (DMSO) as stock solutions and were stored at −20 °C. During analyses using antimycin A (ISN-055) from the NPDepo chemical library, we noticed that ISN-055 contained several different peaks when analyzed by ultraperformance liquid chromatography (Fig. S4*A*). Two other preparations of antimycin A (ISN-060 and FSL0362) that were also purified in the laboratory contained similar peaks, and one of the peaks matched the peak of the commercially obtained pure antimycin A1 (Fig. S4*A*). From the mass spectrometry values of these analyses, other peaks of antimycin A preparations were considered to be from derivatives of antimycin A1 (A2, A3, A4, and A5). The similar activities of these antimycin A derivatives against c-Myc in E-H1 and HCT116 cells were confirmed *via* Western blotting (E-H1 and HCT116 cells) and an INCA assay (E-H1 cells) (Fig. S4, *B* and *C*). The experiments shown in Figures 5, *B* and *D* and 6 were performed using purified antimycin A1.

### Vector construction and infection

Retrovirus vector to express FLAG-tagged c-Myc was constructed from the pMXs retrovirus vector backbone (42) (kindly provided by Prof T. Kitamura). DNA sequence encoding FLAG tag (underscored) containing amino acid sequence (MDYKDDDDKGST) was attached at the N terminus of human c-Myc ORF (WT (encoding UniProt no.: P01106), T58A and S62A mutants). Retrovirus encoding the fluorescent protein Venus (pMXs-Venus) was used as a control. Predicted full sequences of all the vectors are available upon request. A 15:1 mix of the retrovirus vector and a vesicular stomatitis virus-G envelope vector was cotransfected into platE cells (42) (kindly provided by Dr T. Kitamura) by the calcium phosphate method for viral packaging. The collected virus supernatant was infected with HCT116 overnight to obtain a stable cell lines.

### Cell lines

E-H1 and 10-A6 cells (25) were cultured in Dulbecco's modified Eagle's medium (Gibco) with 10% fetal bovine serum (FBS) (Sigma–Aldrich), supplemented with 1% insulin (FUJI FILM Wako Pure Chemical) and 0.5% penicillin–streptomycin (P/S; Invitrogen). The cancer cell lines, HeLa, PANC-1, MIA PaCa-2, DU145, and A549, were cultured in Dulbecco's modified Eagle's medium with 10% FBS and 0.5% P/S, and HCT116 and HL-60 cells were grown in RPMI1640 medium (Gibco) with 10% FBS and 0.5% P/S. They were incubated at 37 °C in a humidified atmosphere containing 5% CO_2_.

### Screening procedure

E-H1 cells (1 × 10^4^ cells/well) were seeded in 96-well plates 1 day before compound addition. One microliter of compounds (original concentration of 2 mM in DMSO, with the hit compound diluted stepwise as needed) with 2.5 μl DOX (8 μg/ml) was added to 200 μl medium and incubated for 24 h. Thereafter, the medium was discarded, and cells were fixed with 200 μl/well of 3.7% formaldehyde in PBS for 15 min, after which the cells were washed with PBS and stained with 100 μl Hoechst 33342 (1 μg/ml in PBS). Subsequently, the fluorescence of EGFP, mKeima, and Hoechst was measured using the setting of FITC, Texas red, and 4′,6-diamidino-2-phenylindole (DAPI), respectively, and INCA and analyzed. Images were quantified using the IN Cell Developer Toolbox 1.9.2 (GE Healthcare).

### Real-time PCR

Real-time PCR analyses of c-Myc mRNA were performed as previously described (25). Primers used for human c-Myc complementary DNA amplification were CAGCAGCGACTCTGAGGAG and GATCCAGACTCTGACCTTTTGC, whereas those used for GAPDH complementary DNA amplification were GAAGGTGAAGGTCGGAGTC and GAAGATGGTGATGGGATTTC.

### Western blotting analysis

E-H1 and cancer cells were seeded at 3 × 10^5^ cells/well in a 6-well plate, cultured with 3 ml of appropriate medium containing 10% FBS per well, and incubated in a 37 °C incubator. After 24 h, the medium was changed, and 1 μl samples were added to 3 ml medium (with or without 100 ng/ml DOX for E-H1 cells) for an appropriate time.

Next, treated cells were washed with 1 ml precooled PBS at 4 °C twice, after which 500 μl of PBS was added to each well, and cells were scraped into a 1.5 ml centrifuge tube and harvested by centrifugation at 5000 rpm for 3 min. Afterward, the cells were lysed in radioimmunoprecipitation buffer and centrifuged at 15,000 rpm for 10 min to collect the supernatant protein lysate. Finally, the protein concentration was quantified using a Pierce BCA Protein Assay kit (Thermo Fisher Scientific).

Cell lysates were separated using SDS-PAGE (5% stacking gel and 7.5% separating gel) and transferred to a polyvinylidene fluoride membrane (Millipore). Next, the membrane was blocked with 5% milk in Tris-buffered saline with Tween for 1 h at 4 °C and incubated overnight at 4 °C with anti-c-Myc (1:1000 dilution; catalog no.: ab32072; Abcam), anti-c-Myc-phosphor T58 (1:1000 dilution; catalog no.: ab185655; Abcam), anti-c-Myc-phosphor S62 (1:1000 dilution; catalog no.: ab185656; Abcam), anti-GSKα/β (1:1000 dilution; catalog no.: 5676; Cell Signaling Technology), anti-GSKα/β-phosphor-S21/9 (1:1000 dilution; catalog no.: 8566; Cell Signaling Technology), or incubated for 2 h at ambient temperature with anti-DYKDDDDK (FLAG) (1:2000 dilution; catalog no.: 014-22383; FUJIFILM), or anti-α-tubulin (1:2000 dilution; catalog no.: T9026; Sigma). The membrane was then washed and incubated with the appropriate secondary horseradish peroxidase–conjugated antibodies for 1 h at 25 °C. After washing, the bound antibodies were detected using SuperSignal West Pico chemiluminescent substrate (Thermo Fisher Scientific) and FUSION SOLO S (Vilber) or using Immobilon-P chemiluminescent substrate (Millipore) and Chemidoc (Bio-Rad). The Western blotting results were quantified using ImageJ software (National Institutes of Health). The membranes were stained with Coomassie brilliant blue to confirm equal loading.

### Mitochondrial ROS detection assay

Mitochondrial ROS levels were measured using a mitochondrial ROS detection assay kit (Cayman Chemical Company), according to the manufacturer’s instructions. E-H1 and HCT116 cells (1 × 10^4^ cells/well) were seeded in 96-well plates 1 day before sample addition. After discarding the medium, 120 μl of cell-based assay buffer was added to each well. Thereafter, the buffer was changed to 100 μl test ROS reagent staining and incubated at 37 °C for 20 min, after which the cells were washed with Hanks' balanced salt solution thrice, and 10 μl of the compound solution was added to 200 μl of Hanks' balanced salt solution. Following incubation at 37 °C for 1 h, the cells were fixed (3.7% formaldehyde, 200 μl/well) for 15 min, washed with PBS, and stained with Hoechst (100 μl of 1 μg/ml in PBS). The fluorescence of ROS reagent staining and Hoechst was measured using FITC (excitation)/Cy3 (emission) and DAPI, respectively, and INCA.

### Cell growth assay

E-H1 and HCT116 cells (1 × 10^4^ cells) were seeded in 96-well plates 1 day before sample addition. Test samples with 2.5 μl DOX (for E-H1 cells; 8 μg/ml) were added to 200 μl medium and incubated for 48 h. Subsequently, the medium was discarded, and cells were fixed (3.7% formaldehyde, 200 μl/well) for 15 min, washed with PBS, and stained with Hoechst 33342 (100 μl of 1 μg/ml in PBS). Finally, the number of nuclei stained with Hoechst dye was measured using DAPI and INCA. In the analyses of the growth inhibition of the cancer cell lines by antimycin A, total cell protein levels were examined after the antimycin A treatment, and the inhibition was determined using DMSO-treated cells as a relative control.

### Analyses of ubiquitination of c-Myc

E-H1 and HCT116 cells were treated with antimycin A, DOX (100 ng/ml), and MG132 (6 μM) for 6 h and lysed in lysis buffer (20 mM Tris–HCl [pH 8.0], 100 mM NaCl, 1 mM EDTA, and 0.5% Nonidet P-40). Next, the cell lysate was incubated with anti-c-Myc-antibody (9B11)-conjugated beads (catalog no.: 3400; Cell Signaling Technology) overnight at 4 °C. Thereafter, the beads were washed thrice with lysis buffer, and the bound proteins were eluted and analyzed using an antiubiquitin antibody by Western blotting.

## Data availability

All data are presented in the article.

## Supporting information

This article contains supporting information.

## Conflict of interest

The authors declare that they have no conflicts of interest with the contents of this article.
