# Supplementary material for: Identification of antimycin A as a c-Myc degradation accelerator via high-throughput screening
Source: J Biol Chem. 2023 Jul 24;299(9):105083. doi: 10.1016/j.jbc.2023.105083 (PMC10470004; doi:10.1016/j.jbc.2023.105083)
Supplement: Supporting Figures S1–S4 [file mmc1.docx]

**Identification of antimycin A as a potent c-Myc degradation accelerator** **via high-throughput screening**

Ziyu Liu, Kosuke Ishikawa, Emiko Sanada, Kentaro Semba, Jiang Li, Xiaomeng Li, Hiroyuki Osada, Nobumoto Watanabe

**Supporting information: Supporting figures 1–4**

**
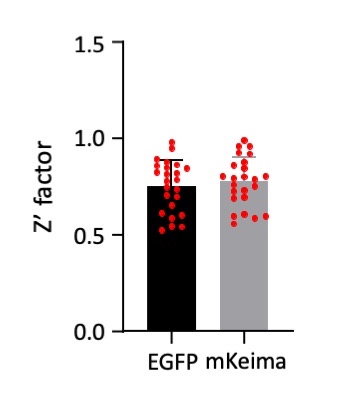
**

**Figure S1. Robustness of the high throughput screening**

Robustness of the high throughput screening (Fig. 3A) was confirmed by the Z’ factors of each plate. Mean of Z’ factors of 24 plates were calculated from EGFP and mKeima expression of positive (with DOX, no compound) and negative (without DOX, no compound) control wells (n=4 each) and shown with SD values. Red dots indicate the individual values of each plate.

**
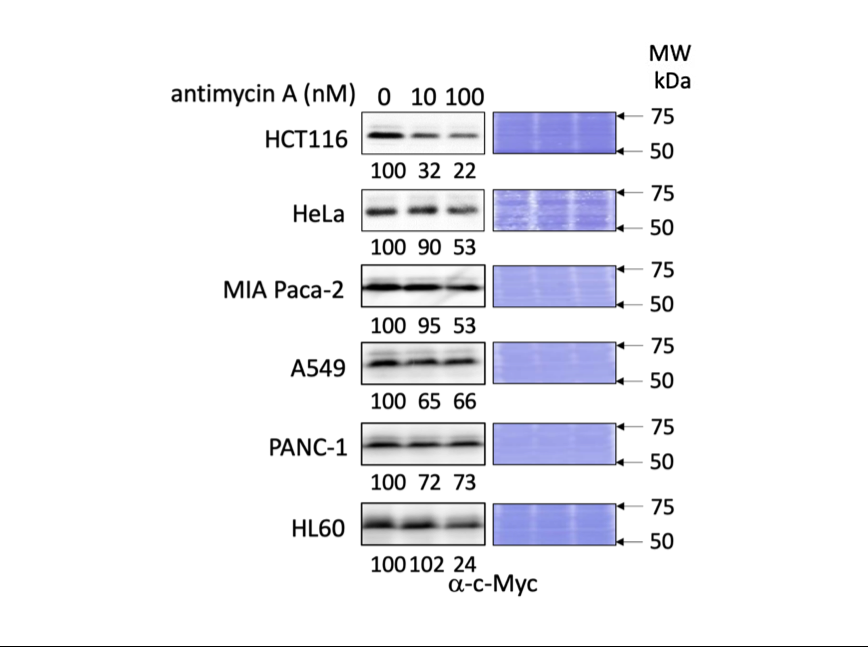
Figure S2. c-Myc protein levels after antimycin A treatment in cancer cells**

Levels of c-Myc protein after antimycin A treatment (10 and 100 nM) in different cancer cells. Cells were harvested and lysed after treatment with antimycin A for 24 h and analyzed by immunoblotting. CBB staining is also shown as a loading control. Quantification of c-Myc levels was performed using ImageJ and shown as the percentage of cells without antimycin treatment. A representative result of two independent experiments is shown. MW, molecular weight.


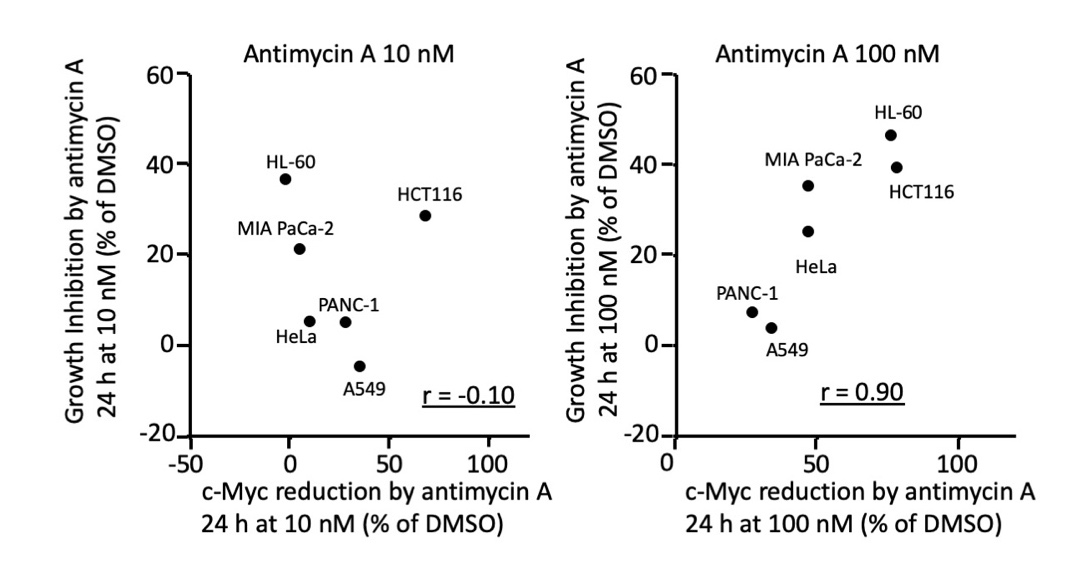


**Figure S3. Correlation between c-Myc reduction and growth inhibition by antimycin A in various cancer cell lines.**

Various cancer cells were treated with antimycin A (left:10 nM, right: 100 nM) for 24 h and c-Myc reduction and growth inhibition was examined in comparison with DMSO treated control cells. c-Myc reduction and growth inhibition were shown as % of control and plotted in X-axis and Y-axis, respectively. A representative result of two independent experiments is shown. The correlation coefficients (r) were calculated and shown in the graph.


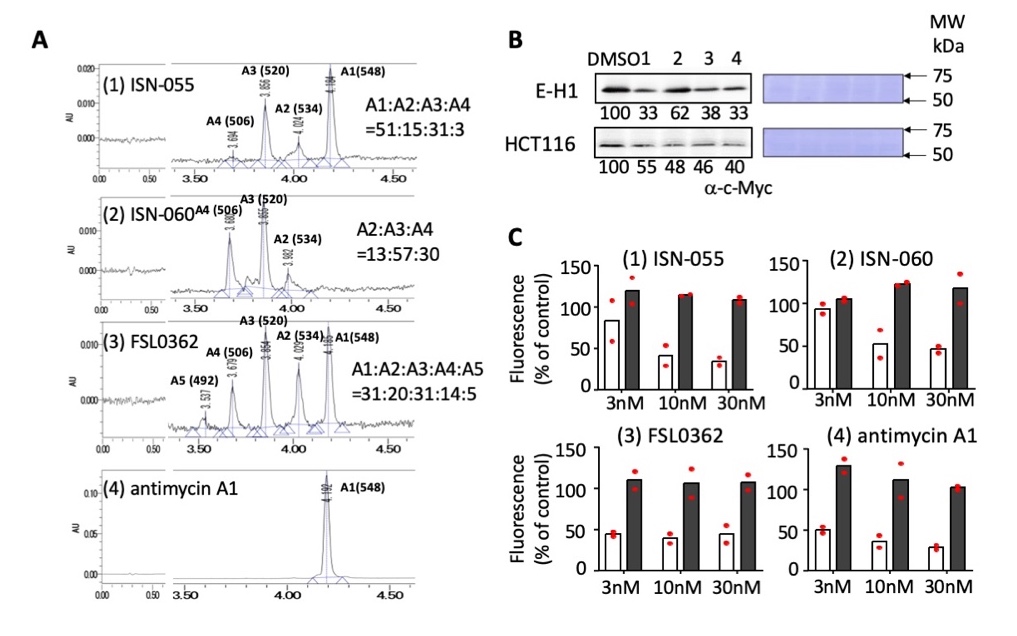
**Figure S4. Analyses of different preparations of antimycin A**

1. UPLC spectra of four preparations: (1) ISN-055, (2) ISN-060, (3) FSL0362, and (4) commercial antimycin A1. The peaks of antimycin A derivatives are shown as the MS values. The ratio of each peak area during preparation is shown in the figure.
2. E-H1 and HCT116 cells were harvested and lysed after treatment with four preparations of antimycin A (1: ISN-055; 2: ISN-060; 3: FSL0362; 4: commercial antimycin A1) at 10 nM for 24 h, and c-Myc degradation was analyzed by immunoblotting. CBB staining is also shown as a loading control. Quantification of c-Myc levels was performed using ImageJ and shown as the percentage of that of the DMSO-treated control. MW, molecular weight.
3. E-H1 cells were fixed 24 h after adding doxycycline (100 ng/mL) and the four preparations of antimycin A [(1) ISN-055; (2) ISN-060; (3) FSL0362; (4) commercial antimycin A1] at the indicated concentrations. The expression of mKeima (closed bar) and EGFP (open bar) in each cell was analyzed using INCA after Hoechst staining. The relative values [% of control (DOX+, without antimycin A)] are shown. A representative of two independent experiments is shown. The red dots indicate the individual measurements.
